# Co-designing interventions to improve emergency department discharge communication with youths, parents and healthcare providers: a process evaluation

**DOI:** 10.1016/j.ijnsa.2025.100362

**Published:** 2025-06-01

**Authors:** Allyson J Gallant, Janet A Curran, Mari Somerville, Lori Wozney, Christine Cassidy, Alannah Delahunty-Pike, Rebecca Mackay, Shannon MacPhee, Emma Burns, Helen Wong, Melanie Doyle, Amy Plint, Roger Zemek

**Affiliations:** a Faculty of Health, Dalhousie University, Room 316-5968 College Street, PO BOX 15000, Halifax, Nova Scotia, NS, B3H 4R2, Canada; b IWK Health Centre, 5850/5980 University Avenue, P.O. Box 9700, Halifax, Nova Scotia, NS, B3K 6R8, Canada; c School of Nursing, Dalhousie University, Halifax, NS, Canada; d CHEO Research Institute, 401 Smyth Road- Research Building 2, 2nd floor, Room 2119, Ottawa, Ontario, ON, Canada, K1H 8L1

**Keywords:** Co-design, Process evaluation, Emergency care, Youth engagement, Parent engagement, Nurse engagement, Physician engagement

## Abstract

**Background:**

Emergency departments (ED) are imperfect environments for information exchange. Communication interventions at discharge can lower readmission rates and improve adherence to follow-up. However, these interventions are rarely designed in partnership with ED clinicians, youth and their parents.

**Objective:**

To describe a theory-based co-design methodology and corresponding process evaluation to improve discharge communication for two common ED presentations: asthma and minor head injury.

**Methods:**

Eligible participants were clinicians who worked in a pediatric ED and parents and youth (aged 12–17) with recent ED experience for either presentation. Co-design teams followed a structured meeting process guided by the Behaviour Change Wheel to facilitate priority setting and intervention design. Process data was captured through meeting recordings, surveys and exit interviews. Quantitative data was analyzed using descriptive statistics and qualitative data through thematic analysis.

**Results:**

Each co-design team included eight members (*n* = 16) participating across eight co-design meetings (mean length: 82 min). The asthma team developed a symptom screening checklist, while the head injury team designed a concussion symptom management tool. Participants reported feeling confident in the co-design process, which increased with active engagement and seeing their decisions incorporated into intervention prototypes. Lengthy meetings and overall time commitment were issues identified by some participants across surveys and interviews.

**Conclusions:**

A theory-based co-design approach provided a useful structure to partner with youth, parents and ED clinicians to develop discharge communication tools. Consideration is needed when scheduling the timing and length of the co-design meetings to account for the schedules of both service providers and users.

**Glossary**
A-EDUCATE: asthma presentation- emergency department discharge communication strategies
APEASE: acceptability, practicability, effectiveness, affordability, safety, equity
BCT: behaviour change technique
BCW: behaviour change wheel
ED: emergency department
KT: knowledge translation
M: mean
MHI: minor head injury
MHI-EDUCATE: minor head injury presentation- emergency department discharge communication strategies
OECD: Organization for Economic Co-operation and Development
SD: standard deviation
UX: user experience

**Contribution of the paper**

What is already known about the topic?

• Co-design literature has grown in recent years, but details regarding the procedures are often atheoretical, inadequately described and rarely evaluated.
• Despite youth being the recipients of many health interventions, they are rarely included in the intervention design process in meaningful ways.

What this paper adds

• Using the Behaviour Change Wheel, we detail and evaluate an eight-meeting co-design structure with youth, parents and ED clinicians
• We provide researchers with a comprehensive description of effective methods to use in co-designing and evaluating future health interventions with a range of health system stakeholders.

Alt-text: Unlabelled box

## 1 Background

Emergency departments (EDs) are experiencing unprecedented strain, with record wait times, staff shortages and closures in recent years (CADTH Health Technology Review Recommendation, 2023). For example, over 2.5 million pediatric patients (aged 0–19 years) attended ED in Canada in 2021–2022, while data from Organization for Economic Co-operation and Development (OECD) countries consistently highlight the overuse, and often misuse, of EDs (Berchet, 2015; Canadian Institute for Health Information, 2022; Goiana-da-Silva et al., 2025). Given the chaotic ED environment, the discharge communication process is often fragmented and can result in miscommunications between clinicians (i.e., nurses and physicians), pediatric patients, and their parents (i.e., parents, caregivers or legal guardians; Samuels-Kalow et al., 2016, 2013). Comprehension of what occurred during the ED visit and care required at home is vital for the safe transition of care to home (Curran et al., 2019). A range of communication interventions are being developed and tested to ensure parents adequately and accurately understand their children’s diagnosis, medications administered, and follow-up care plans from their clinicians upon ED discharge (Wozney et al., 2022).

Partnering with parents, youth and clinicians in health system redesign can improve care and patient outcomes (Johnson et al., 2008). A co-design approach- an iterative, interactive, and inclusive process which generates new solutions by actively engaging stakeholders- is necessary to address these health system gaps (Curran et al., 2023; Stacey and Wilson, 2014). These methods have been used with patients and families to manage prediabetes and perinatal care, and can support intervention uptake and sustainability (Farao et al., 2020; Reid et al., 2022; Tay et al., 2021). However, youth and parent engagement in co-designing healthcare interventions is often limited (Crowther et al., 2023). While there are a range of benefits to co-design, there are currently limited health research studies which use these methods, and many are atheoretical and lack sufficient detail to promote replication and evaluation (Slattery et al., 2020).

**Fig. 1:**
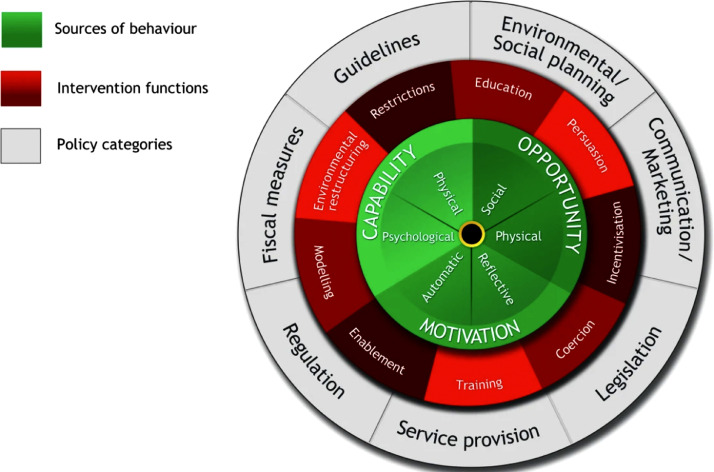
The behaviour change wheel (Michie et al., 2014).

Using theory to support intervention design has been associated with improved intervention effectiveness (Skivington et al., 2021). A range of tools have been developed to facilitate theory application in intervention design, including the Behaviour Change Wheel (BCW- Fig. 1), behaviour change techniques (BCTs) and the acceptability, practicality, effectiveness, affordability, safety and equity (APEASE) criteria. The BCW is a synthesis of 19 behaviour change frameworks distilled into three phases to support the systematic identification of determinants to target behaviours, which then map to specific intervention functions and policy components to effectively address the source(s) of target behaviours (Michie et al., 2014). The BCTs offer a range of ‘active ingredients’ to further inform BCW intervention functions, while the APEASE criteria provides evaluation criteria to consider throughout the BCW process (Michie et al., 2014). Using the BCW and complementary tools allows co-design teams to follow a transparent and systematic approach throughout intervention design and has been widely used to inform clinical and public health interventions (Cassidy et al., 2017; Gallant et al., 2024; Gould et al., 2017).

Previous research has identified a lack of evaluation procedures to determine the success of co-design methods (Peters et al., 2024; Slattery et al., 2020). Process evaluation is an effective approach to assess health system redesign in partnership with knowledge users who bring varying perspectives to the evaluation (Saunders et al., 2005). This type of evaluation can provide important contextual information about how and why a process (such as co-design) was or was not effective (Jepson et al., 2022). Conducting a process evaluation would offer valuable insights into the experiences of different stakeholder groups involved in intervention co-design.

As most pediatric ED patients are discharged home (Canadian Institute for Health Information, 2024), co-designing discharge communication interventions with youth, their parents and clinicians presents an opportunity for these partners in care to actively inform the design of interventions to meet their specific needs. Co-designing healthcare services in this context can challenge existing hierarchies within the health system that represent system-centred rather than person-centred design (Curran et al., 2020). The overall aim of this study was to operationalize a theory-based co-design method with youth, parents and ED clinicians to develop two **e**mergency department **d**ischarge comm**u**ni**c**ation str**ate**gies (EDUCATE): one for asthma presentations (A-EDUCATE) and one for minor head injury presentations (MHI-EDUCATE). Here, we present the co-design procedures and process evaluation findings.

## 2 Methods

### 2.1 Study design

**Fig. 2:**
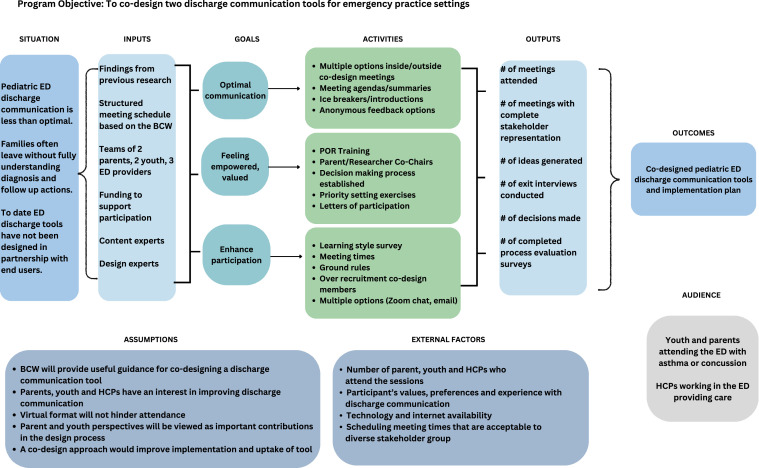
Educate logic model. Abbreviations: BCW: behaviour change wheel; ED: emergency department; HCP: healthcare provider; POR: patient-oriented research.

The EDUCATE study protocol provides additional methodological insights (Curran et al., 2020). A logic model outlining the study components to guide the process evaluation is provided in Fig. 2. This study was supported by Pediatric Emergency Research Canada (PERC) and ethics approval was obtained from the IWK Health Centre Research Ethics Board (REB; Reference: 1024,004) and the Children’s Hospital of Eastern Ontario (REB: 21/62X).

### 2.2 Co-Design team composition- eligibility & recruitment

Each EDUCATE co-design team was comprised of youth, parents and ED clinicians who were recruited through an academic, pediatric tertiary hospital in Canada which sees over 42,000 ED patients annually (Nova Scotia Government, 2023). Maximum variation sampling was used to enroll stakeholders across a range of socio-demographic characteristics to diversify participants within and across both co-design teams (Nyimbili and Nyimbili, 2024). We also recruited multiple participants per stakeholder group to account for potential absenteeism.

Clinicians were eligible to participate if they regularly worked shifts in the ED. They were recruited through targeted email invitations, and interested clinicians provided informed, written consent prior to completing a socio-demographic questionnaire in Research Electronic Data Capture (REDCap; Harris et al., 2009).

Parents and youth were eligible to participate if they had recently attended an ED with an asthma or MHI presentation and spoke English as a primary or additional language. Youth were required to be between the ages of 12–17 to participate and could participate as a youth-parent dyad where preferred. These participants were recruited through social media posts and study posters placed in the ED. Interested youth and parents provided informed, written consent and completed the socio-demographic screening questionnaire (e.g., gender, age category) online in REDCap (Harris et al., 2009). The Medical Term Recognition Test (METER; Rawson et al., 2010) is a health literacy scale initially included in the questionnaire, but an error in display logic resulted in incomplete data. Therefore, METER scores could not be reliably calculated and this data was excluded from the study.

### 2.3 Research team composition

Each EDUCATE co-design teams included a team of researchers, including the lead researcher and parent partner, knowledge translation (KT) experts (*n* = 2), a human performance technology expert (*n* = 1), research coordinators (*n* = 2), trainees (*n* = 2), content experts (*n* = 2), and user experience (UX) designers (*n* = 2). An undergraduate nursing student (*n* = 2) tracked decisions made, participant contributions and engagement across meetings for each team.

### 2.4 Co-design meeting procedures

All meetings were co-facilitated by the lead researcher and a parent partner. We originally proposed hosting six two-hour meetings with each co-design team over 12 months to develop and refine intervention prototypes. However, the COVID-19 pandemic public health measures resulted in hosting and recording the meetings online using Zoom Video Communications (Zoom Video Communications Inc., San Jose, USA). Participants were asked to leave their cameras and microphones on during the meeting and could also use the chat feature to share ideas. The switch to online meetings resulted in eight 90-minute meetings to accommodate for the fatigue that can occur with extended online meetings (Archibald et al., 2019). The planned objectives tied to the original six meetings were adjusted to be covered across the eight online meetings. Both the A-EDUCATE and MHI-EDUCATE team meetings followed the same schedule based on the BCW to identify important behavioural targets and guide intervention development and decision-making, as described below (Michie et al., 2014). Meetings were audio-recorded in Zoom, with files downloaded and securely stored following each meeting.

#### 2.4.1 Pre-meeting procedures

The lead researcher and parent partner met to co-develop the meeting agendas. The lead researcher also met with content and methods experts to review the agendas and to align specific meeting goals with their planned activities for the co-design teams.

The research coordinator sent a meeting reminder one week before each scheduled meeting, followed by a detailed agenda, supporting documents and Zoom login details two days before the meeting. Questions about the meeting or those unable to attend were noted by the research coordinator. The research team received annotated versions of the agendas one week in advance, which outlined the purpose of each activity and team member’s roles and responsibilities during the meeting.

#### 2.4.2 Meeting procedures

##### 2.4.2.1 Orientation meeting

All co-design and research team members attended an online orientation session in July 2020. A patient engagement coordinator gave a 30-minute presentation of the importance of patient-oriented research. The lead researcher then reviewed the overall aim for the study and worked with the co-design teams to develop specific timelines for the intervention design meetings (e.g., preferred days/ times to meet). Co-design team members were invited to complete a needs assessment survey in REDCap to identify preferred learning styles and methods (e.g., lectures, discussions, webinars) to incorporate into the meetings.

##### 2.4.2.2 Intervention design meeting 1

The first co-design meeting included a 30-minute presentation by a content expert to provide an overview of the illness presentation (i.e., asthma or MHI), standard care provided during an ED visit and usual discharge communication instructions. Co-design team members then brainstormed a range of priority target behaviours that could be addressed in an A-EDUCATE or MHI-EDUCATE intervention.

##### 2.4.2.3 Intervention design meeting 2

The KT experts participated in Meeting 2 which included three key activities. First, the co-design team reviewed the list of potential targets generated during Meeting 1 and added any additional target behaviours considered between meetings. Second, the team undertook a priority setting exercise to rank their individual top five target behaviours through an online poll. Third, they brainstormed the potential barriers to engaging in each of the top five behaviours.

##### 2.4.2.4 Intervention design meeting 3

The KT experts also participated in Meeting 3 to guide refinement of each intervention’s focus. After reviewing the barriers to addressing target behaviours generated in Meeting 2, teams worked with the KT experts to consider each of the five behaviours against the APEASE criteria (Michie et al., 2014). A consensus voting process was used to select the priority target behaviour to move forward with intervention design.

##### 2.4.2.5 Intervention design meeting 4

The KT experts and a human performance technology expert worked with the co-design teams during Meeting 4. The group activities included storyboarding potential content and intervention prototypes using the BCW and BCTs. For example, the education BCW intervention function could map with the ‘instruction on how to perform the behaviour’ BCT (Michie et al., 2013). Following the identification of appropriate BCW and BCT content, the human performance technology expert worked with co-design teams to identify potential modes of delivery for the intervention. Each identified mode of delivery was considered against the APEASE criteria to select the proposed intervention’s content and format. Following Meeting 4, final decisions were shared with the UX designers to develop intervention prototypes.

##### 2.4.2.6 Intervention design meeting 5

The UX designers used Meeting 5 to present two intervention prototype options with key design features to each co-design team. The co-design teams discussed each prototype to garner feedback and compared each prototype against the APEASE criteria. A consensus vote was held to identify the preferred prototype to move forward to development, which the UX designers then built. This developed prototype was used for the first round of usability testing (Somerville et al., 2025). Usability testing participants were from relevant stakeholder groups but did not include co-design members as participants in this phase of work.

##### 2.4.2.7 Intervention prototype refinement meeting 6

The co-design teams were presented with the findings from usability testing round 1. The team discussed the findings and used a process of consensus to determine required prototype revisions. Suggested revisions were made by the UX designers prior to usability testing round 2.

##### 2.4.2.8 Intervention prototype refinement meeting 7

Co-design teams were presented with the findings from usability testing round 2. Co-design teams discussed the feedback and again used a process of consensus to develop recommendations for further tool refinement. The UX designers updated the prototypes, which were circulated to the co-design teams by email for any final feedback.

##### 2.4.2.9 Intervention prototype celebration meeting 8

Co-design teams were presented with final tool prototypes in Meeting 8. All co-design team members received a letter of participation and a token of appreciation to mark the conclusion of the study.

### 2.5 Post-meeting procedures

#### 2.5.1 Process evaluation survey

Attendees were asked to complete a process evaluation survey in REDCap after each meeting. Survey data were compiled and de-identified by the research coordinator. Co-design team members received a $30 gift card as an honorarium for each meeting attended and evaluation survey completed.

#### 2.5.2 Communication with co-design teams

The research coordinator circulated a summary of decisions made to co-design team members following each meeting. Emails were also sent to co-design team members 4–6 weeks in advance of the next planned meeting to provide study updates and to determine availability for the next meeting. As there were longer time periods between prototype development meetings for usability data collection, the research coordinator emailed team members bimonthly to provide updates on data collection and study progress.

#### 2.5.3 Communication within the research team

Core research team members met biweekly to (1) discuss overall study status; (2) debrief on the recent co-design meetings; (3) discuss data collection and preliminary findings; and (4) troubleshoot any identified issues. The research team also held regular meetings with the UX designers to guide intervention prototype development. Asthma and MHI content experts were included in these meetings to confirm essential content to include in each intervention.

### 2.6 Post-study procedures

Team members were invited to participate in an exit interview to share their experiences during the co-design process. A semi-structured interview guide was developed to elicit feedback on the theory-based structure of the meetings, their experiences on the co-design team, and barriers and enablers to participation in the co-design methods. All interviews were conducted via Zoom or telephone by a trained researcher who was not in attendance at the co-design meetings to reduce potential bias.

### 2.7 Data analysis

Process evaluation survey data were analyzed using frequency counts and descriptive statistics in Microsoft Excel. Meeting recordings were reviewed to confirm decisions were captured accurately. Exit interview recordings were independently analyzed using inductive thematic analysis by a trainee to identify key themes, which were verified by a second reviewer (Braun and Clarke, 2012).

## 3 Results

### 3.1 Meeting and participant characteristics

The A-EDUCATE team comprised: one ED nurse, two ED physicians, two parents, one youth, and one parent-youth dyad. One youth participated as a near-peer to enhance the youth perspectives on asthma ED discharge communication. All co-design team members identified as female and were new to collaborative, patient-oriented research.

The MHI-EDUCATE team comprised: three ED nurses, one ED physician, two parents, one youth, and one parent-youth dyad. One team member identified as gender fluid, while the remaining participants identified as female. All MHI co-design team members also noted this was their first experience with patient-oriented research.

The orientation meeting was held in July 2020 and lasted 115 min. Both teams held their respective eight meetings between September 2020- December 2022. The A-EDUCATE meeting length ranged from 26–114 min (mean[m]: 82 min; standard deviation [SD]: ± 31.18), while MHI-EDUCATE meetings ranged from 25–115 min (m: 82 min; SD: ± 35.47). Clinician, parent and youth perspectives were represented at each co-design meeting, and participants equally contributed to generating ideas and decision making across the eight meetings.

### 3.2 Meeting decision making

**Fig. 3:**
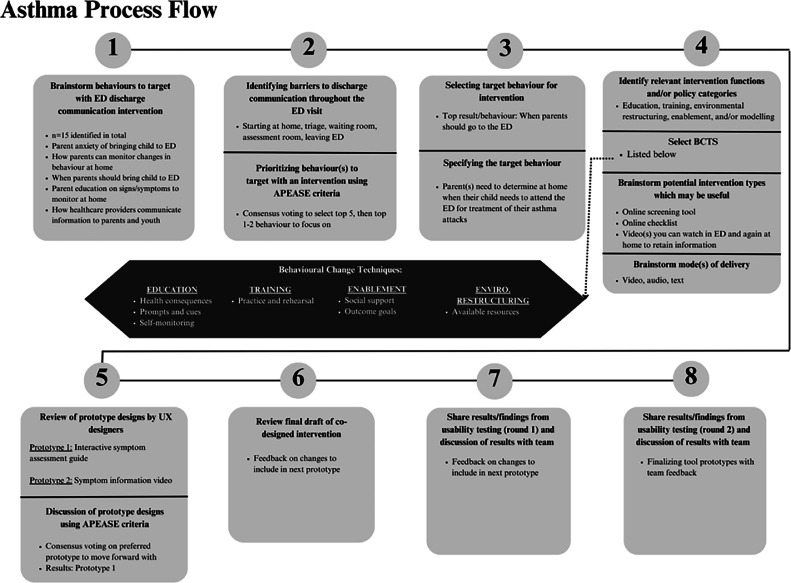
A-EDUCATE Process.

**Fig. 4:**
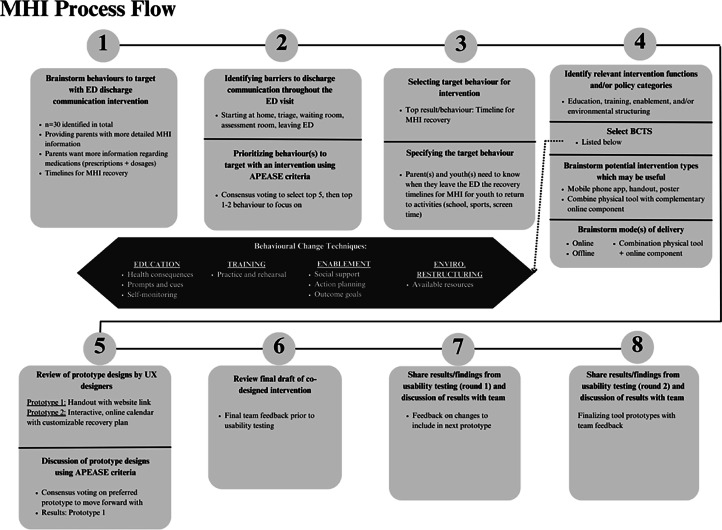
MHI-EDUCATE Process.

A summary of A-EDUCATE and MHI-EDUCATE intervention decisions and prototype refinements can be found in Fig. 3, Fig. 4 below.

#### 3.2.1 Orientation meeting

Participant surveys identified a preference for discussions (*n* = 11/14; 79 %), case studies (*n* = 9/14; 64 %) and synchronous webinars (*n* = 8/14; 57 %). Participants also noted holding co-design meetings during weekday evenings was preferred for their schedules. These findings were incorporated into the meeting structure, where possible.

#### 3.2.2 Intervention design meeting 1

A-EDUCATE brainstorming identified 15 potential ED discharge communication behaviours to target with an intervention, while MHI-EDUCATE identified 30 potential behaviours. Example behaviours can be seen in Fig. 3, Fig. 4.

#### 3.2.3 Intervention design meeting 2

**A-EDUCATE:** The first round of priority setting consensus voting identified five target behaviours to include in the next activity: 1) ensuring parents/families understand asthma medication administration; 2) parents/youth uncertainties of when to attend the ED during an asthma attack; 3) parents/families having a better understanding of asthma signs and symptoms; 4) understanding potential asthma triggers; and 5) understanding the acute and chronic nature of an asthma diagnosis. Barriers identified across these behaviours included: parental stress, language barriers, medication costs, physicians’ overestimating parent/patient knowledge, and lack of time and resources for adequate counselling on chronic disease management.

**MHI- EDUCATE:** The five identified target behaviours to consider were: 1) education resources on MHI and levels of severity; 2) knowing when to return to the ED; 3) what to expect at home following MHI; 4) monitoring changes in behaviour following MHI; and 5) MHI recovery timelines. Identified barriers to these behaviours included: evolving nature of MHI symptoms, inadequate information provided during an ED visit, and inconsistencies between clinician’s advice and handout materials.

#### 3.2.4 Intervention design meeting 3

**A-EDUCATE:** The priority setting voting resulted in selecting ‘parents/youth uncertainties of when to attend the ED during an asthma attack’ to address with an intervention. Specific barriers to address included knowledge of asthma, parent and youth anxiety, and supporting parental assessment and youth self-assessment of an asthma attack.

**MHI-EDUCATE:** The priority setting vote identified a desire to design an intervention to outline an MHI recovery and return to activities timeline, with a specific focus on concussion recovery. Specific barriers included the complexities of concussion healing, inconsistencies between clinician’s advice and education resource details, and what activities are safe to engage in during concussion recovery.

#### 3.2.5 Intervention design meeting 4

**A-EDUCATE:** BCW intervention functions selected to inform prototype development were: education, training, environmental restructuring, enablement and modelling. BCTs associated with these intervention functions included: *information about health consequences, self-monitoring of behaviour, feedback on outcomes of behaviour* and *adding objects to the behaviour*. The team decided on a combination of video, audio, and/or handouts for intervention delivery. Discussions were also held to ensure the modes of delivery would be accessible for patients of different cultural and health literacy backgrounds.

**MHI-EDUCATE:** Key intervention functions to include in the concussion intervention prototype were: education, training, enablement, and environmental restructuring. BCTs considered for the intervention prototype included: *self-monitoring of behaviour, feedback on behaviour, review outcome goals,* and *prompts/cues.* The team selected a combination of handouts and/or website delivery. As with A-EDUCATE, the team ensured the intervention would be accessible for patients of different cultural and health literacy backgrounds.

#### 3.2.6 Intervention design meeting 5

**A-EDUCATE:** The UX designers developed two intervention prototypes: 1) an asthma symptom screening checklist handout; and 2) an asthma informational video, which could be viewed in the ED prior to discharge and at home. Prototype 1 used a traffic-light colour system to track symptom severity. It would involve a slideshow or gallery of information on different symptoms, with the potential to build into a website with interactive symptom tracker and used to share information with other caregivers (e.g. teachers, babysitters). Prototype 2 included demonstrations of medication administration and symptoms that would prompt an ED visit. The video would help patients evaluate the severity of symptoms, showing what concerning symptoms may look or sound like (e.g., tracheal tug, wheezing). The video would provide education on symptom progression and avoiding asthma triggers.

Team consensus voting resulted in Prototype 1 being selected for further design and usability testing. Through team discussions, adding more visuals to the checklist and making the checklist available online were suggested to enhance accessibility for patients with language or literacy barriers.

**MHI-EDUCATE:** The UX designers presented two intervention prototypes: 1) a concussion symptom management handout with complementary website; and 2) an online concussion recovery plan. Prototype 1 included a paper handout with a high-level overview of concussion symptoms and recovery. The handout would provide a link to a website via a QR code or URL link. The complementary website would include audio clips and information to be shared with other caregivers (e.g., coaches, babysitters). Prototype 2 included an interactive calendar with a customizable concussion recovery plan. The planner would provide timelines and milestones for a typical recovery and include a tailored plan that was discussed with the family (e.g., return to sports, screentime).

The team voted to move forward with Prototype 1 for design and usability testing. There was no significant team feedback to revise the prototype prior to testing.

#### 3.2.7 Intervention prototype meeting 6

Usability testing round 1 identified minor issues with the A-EDUCATE and MHI-EDUCATE prototypes (Somerville et al., 2025). Resolution ideas generated by each team were provided to the UX designers to incorporate into the next prototype iterations.

#### 3.2.8 Intervention prototype meeting 7

Team discussions were held to discuss usability testing round 2 results and ideas for improving each prototype. The A-EDUCATE team suggested adding additional information pages with a brief outline describing asthma and medication details (e.g., different inhalers for different age groups). The MHI-EDUCATE team feedback was mostly cosmetic but included adding more specific details around certain symptoms (e.g., frequency of vomiting episodes within a 24-hour period).

#### 3.2.9 Intervention prototype meeting 8

Results from usability testing round 2 were presented to the clinicians, youths and parents. The final prototypes were shared with co-design team members.

### 3.3 Process evaluation surveys

Over two-thirds (11/16; 69 %) of participants completed the process evaluation survey following each meeting (range: 6/16 [38 %] in meeting 1 to 14/16 [88 %] for meetings 4 and 5). MHI-EDCUATE participants were consistently more likely to complete the survey compared to A-EDUCATE members. Results indicated participants across stakeholder groups had positive experiences during each EDUCATE meeting and with the overall project (Table 1). Participants reported feeling moderately comfortable presenting their thoughts during earlier intervention design meetings (m: 4.91/7; SD: ±2.59), however mean scores from the remaining meetings were high (mean ranges: 5.29–6.67/7). The length of design meetings was a challenge noted from the open-ended survey responses. Solutions suggested by clinicians included having longer meetings (e.g., 2 h) with a break, or shorter, but more frequent, meetings during the study.

**Table 1:** Summary of Process Evaluation Data Across Meetings.

**Survey Item**	**Meeting #**							
	**1 (*n*=6; 2 MHI and 4 A)**	**2 (*n*=11; 7 MHI and 4 A)**	**3 (*n*=13; 8 MHI and 5 A)**	**4 (*n*=14; 8 MHI and 6 A)**	**5 (*n*=14; 8 MHI and 6 A)**	**6 (*n*=13; 8 MHI and 5 A)**	**7 (*n*=8; 5 MHI and 3 A)**	**8 (*n*=7; 4 MHI and 3A)**
Did you feel prepared to participate in the discussion?	Yes (*n* = 6)	Yes (*n* = 10) Unsure (*n* = 1)	Yes (*n* = 13)	Yes (*n* = 14)	Yes (*n* = 13) No (*n* = 1)	Yes (*n* = 13)	Yes (*n* = 8)	Yes (*n* = 7)
Did you feel you had the materials you needed prior to attending this meeting?	Yes (*n* = 5) No response (*n* = 1)	Yes (*n* = 11)	Yes (*n* = 13)	Yes (*n* = 14)	Yes (*n* = 14)	Yes (*n* = 12) No (*n* = 1)	Yes (*n* = 8)	Yes (*n* = 7)
Did you feel supported to participate to the extent that you would have liked in the meeting today?	Yes (*n* = 6)	Yes (*n* = 11)	Yes (*n* = 13)	Yes (*n* = 14)	Yes (*n* = 14)	Yes (*n* = 13)	Yes (*n* = 8)	Yes (*n* = 7)
Do you understand where you can get support to address any concerns you have with your participation in this project?	Yes (*n* = 6)	Yes (*n* = 11)	Yes (*n* = 12) No (*n* = 1)	Yes (*n* = 13) Unsure (*n* = 1)	Yes (*n* = 14)	Yes (*n* = 13)	Yes (*n* = 8)	Yes (*n* = 7)
Do you understand where you can find additional information about the project?	Yes (*n* = 6)	Yes (*n* = 10) Unsure (*n* = 1)	Yes (*n* = 11) Unsure (*n* = 2)	Yes (*n* = 12) Unsure (*n* = 2)	Yes (*n* = 14)	Yes (*n* = 13)	Yes (*n* = 8)	Yes (*n* = 7)
How comfortable are you with your understanding of the purpose of the project? (score range: 1= very uncomfortable; 7= very comfortable)	mean: 6.5; SD: ±0.55	mean: 5.09; SD: ± 2.66	mean: 6.00; SD: ±1.58	mean: 5.79; SD: ± 2.08	mean: 6.00; SD: ± 1.65	mean: 6.77; SD: ±0.44	Mean:6; SD: ± 2.24	mean: 4.43; SD: ±3.21
How comfortable did you feel presenting your ideas, experiences, and opinions today? (score range: 1= very uncomfortable; 7= very comfortable)	mean: 6.67; SD: ±0.52	mean: 4.91; SD: ± 2.59	mean: 6.62; SD: ± 0.51	mean:6.29; SD: ± 1.59	mean: 5.71; SD: ± 2.05	mean: 6.54; SD: ± 0.66	mean:6; SD: ±2.07	mean:5.29; SD: ± 2.93
In your opinion, have your insights and comments impacted the decisions made during this meeting?	Yes (*n* = 4) No (*n* = 1) Unsure(*n* = 1)	Yes (*n* = 11)	Yes (*n* = 13)	Yes (*n* = 14)	Yes (*n* = 14)	Yes (*n* = 11) Unsure (*n* = 2)	Yes (*n* = 8)	Yes (*n* = 6) Unsure (*n* = 1)
In your opinion, was the content that was presented today relevant to the tasks of the project ?	Yes (*n* = 6)	Yes (*n* = 11)	Yes (*n* = 13)	Yes (*n* = 14)	Yes (*n* = 14)	Yes (*n* = 13)	Yes (*n* = 8)	Yes (*n* = 7)
In your opinion, was the content presented today clear?	Yes (*n* = 6)	Yes (*n* = 11)	Yes (*n* = 13)	Yes (*n* = 14)	Yes (*n* = 14)	Yes (*n* = 13)	Yes (*n* = 8)	Yes (*n* = 7)
In your opinion, was the length of time scheduled for this meeting sufficient?	Yes (*n* = 5) Unsure(*n* = 1)	Yes (*n* = 10) No (*n* = 1)	Yes (*n* = 11) No (*n* = 2)	Yes (*n* = 14)	Yes (*n* = 14)	Yes (*n* = 12) No (*n* = 1)	Yes (*n* = 8)	Yes (*n* = 7)

### 3.4 Exit interviews

Exit interviews were conducted with eight participants, consisting of six from the A-EDUCATE and two from the MHI-EDUCATE teams. Two participants from each stakeholder group participated (i.e., two nurses, two physicians, two parents, and two youths), with interviews ranging from 5–11 min (m: 8.75; SD: ±1. 98). Overall, participants’ responses corroborated process evaluation survey themes. Participants felt the BCW was an accessible tool to use in the co-design methods by expediting decision-making, prioritizing ideas and allowing for meetings to advance rather than getting caught up in discussions and stalling progress. A strength recognized by several participants was having the meetings facilitated to encourage equal participation across stakeholder groups. Participants felt their feedback and ideas were actively considered and incorporated into the intervention development and refinement, and appreciated being involved throughout the entire design process:

*“I think it was very user-centered which was really good as opposed to other experiences, through work or other experiences, where it’s like ‘here’s the design- should we change the colour?’ [laughs]. Here, we were brought in at the beginning, through the journey, and the final product as a result of it.”* (parent)

Including youth in the co-design process was specifically highlighted as a benefit of this study by the ED nurses and physicians as it was an important, but often overlooked, view when designing tools for this population:

*“I think having the youth participate and getting a chance to hear their experiences and what they thought was valuable… And I think because it carried on over a couple of years, watching their involvement grow over time was really interesting.”* (physician)

Youth also highlighted they felt engaged and involved throughout the co-design process. Having more than one youth on each team was considered a benefit as it helped them feel more confident expressing their thoughts and feedback:

*“Having peers there was real helpful. I think at the beginning I…was like is this only going to be highly qualified, really educated adults? I would have felt more hesitant speaking up and joining in.”* (youth)

While co-design methods were viewed positively overall, some challenges were noted. Primarily it was difficult to have meetings where all participants could attend. Participants also noted they would like to get an update on the use of the tools after the study to see the real-world outcomes from the work they helped produce:

“*… it would be nice to hear about how it’s used… its application real life, if it’s used in real life, like 6 months down the road or a year or what was the results”* (parent)

## 4 Discussion

This study developed and evaluated co-design methods including clinicians, parents, and youths for two pediatric ED discharge communication interventions. Each co-design team included eight participants attending eight meetings over two years to improve the transition in care from ED to home for asthma and MHI presentations. Process data indicated the co-design methods and study involvement was a positive experience for participants, with challenges reported related to the length of meetings and time commitment to participate by some participants. Both co-design teams followed the same theory-based procedure and were successful with developing discharge communication interventions, which are now ready for future feasibility testing in an ED setting.

We identified several benefits of actively engaging clinicians, parents, and youth in the structured co-design method, including cohesive participation across stakeholder groups and their representation at each meeting. This is consistent with other co-design research using the BCW with parents, youth and health experts to co-design health interventions (Creaser et al., 2023). Considerable effort from the research team went into planning meeting times and resources that worked for three stakeholder groups with competing schedules, demands and priorities. The time commitments and significant resources required to conduct meaningful co-design research is well established (Ní Shé and Harrison, 2021; Noorbergen et al., 2021; Slattery et al., 2020); Future research teams should value and prioritize the time commitment required, often outside typical office hours, to actively engage a range of stakeholders in co-design research. Focused discussion clarifying the time commitments required from participants to engage across the full co-design process as well as alternative strategies for participation is necessary at the outset and is a critical aspect of the process evaluation of co-design methods (Kiss et al., 2024).

Youth participants on the EDUCATE co-design teams spoke about how the experience gave them more confidence to participate in meetings among parents, clinicians and researchers. However, youth participation during the meetings varied, with some youths needing prompting to share their opinions during decision-making points. We alternated between discussions or online anonymous voting strategies (e.g., polling) to support participation in decision-making by youth who were less inclined to contribute. Research has highlighted the unique ways youths use online (e.g., social media) and ‘offline’ engagement in their daily lives, and these skills could be used to support involvement in health and public decision-making (Crowley and Moxon, 2018; Willems et al., 2012). Future co-design research that includes a range of knowledge users and providers may benefit from specifically identifying youths’ communication preferences prior to study commencement to ensure youths are actively engaged in decision-making in ways meaningful to them throughout the research study.

While we cannot yet speak to the impact of our interventions in the real-world, our approach of including ED service users (i.e. youth and parents) and service providers (i.e., clinicians), in outlining clear co-design methodological details and conducting a process evaluation helps address many of the established gaps in co-design research literature. Reviews of co-design methods and processes have consistently identified a lack of reporting clarity, varied definitions and approaches to co-design, and minimal evaluation of the methods or interventions designed (Chinsen et al., 2025; Kiss et al., 2024; Slattery et al., 2020). Co-design research has grown substantially in recent years (Chinsen et al., 2025), and the potential benefits for tailoring interventions to the specific needs and context of the target population is well documented (Talevski et al., 2020). Yet, there is dearth of literature on the effectiveness of these interventions compared to traditional intervention design approaches (Chinsen et al., 2025; Slattery et al., 2020). While this study addresses some of the key deficits in co-design research literature, further evaluation work is needed to measure the impact of co-designed interventions (Alpeza et al., 2024.; Zacharuk et al., 2024). We argue well-designed effectiveness-implementation hybrid studies are needed to compare the effectiveness of co-designed and traditional interventions and their implementation strategies to build out the co-design evidence base. The recently developed Co-Design Evaluation Framework offers a guide for researchers to inform these studies, their processes and impacts (Peters et al., 2024).

Strengths of this study include actively engaging youths, parents and ED clinicians in intervention design, using the BCW to guide theory-driven methods, and collecting quantitative and qualitative process data to evaluate participants’ experiences with the co-design process. Limitations include challenges with recruiting youth, resulting in a near peer participant whose perspectives may differ from true lived experience. The shift to online meetings may have facilitated meeting attendance, particularly for ED clinicians, but may have added burden to meeting length and frequency. Additionally, all co-design team members were recruited from one ED site in one Canadian province and were predominantly female; therefore, the interventions designed are specific to the needs of this ED and these participants. Target behaviours identified and interventions designed may vary based on co-design team composition, target illness presentation(s), and/or EDs included. While our co-design teams were cognizant that interventions designed were functional for vulnerable and equity-seeking groups, diversifying recruitment across gender, ethnicity, languages and/or literacy levels would be beneficial in future studies to ensure interventions designed are actively meeting the unique needs of these populations. Despite these limitations, we have developed and evaluated a successful approach to co-designing interventions which can be utilized by researchers in future health intervention studies.

## 5 Conclusions

This study implemented a process evaluation to explore youth, parent and clinician participation in a theory-based co-design procedure to develop an ED discharge communication intervention. Overall, participants across all stakeholder groups reported a positive experience with the co-design procedure and seeing their contributions inform the design of A-EDUCATE and MHI-EDUCATE interventions. Co-design teams would benefit from early discussions with participants about time commitment expectations to engage in the co-design process as well as possible alternatives for contributing when attending in-person meetings is not possible.

## Funding

This study was funded by the Canadian Institutes of Health Research, Project Grant (Application #399,798).

## CRediT authorship contribution statement

**Allyson J Gallant:** Writing – review & editing, Writing – original draft, Project administration, Investigation, Formal analysis, Data curation. **Janet A Curran:** Writing – review & editing, Methodology, Investigation, Funding acquisition, Data curation, Conceptualization, Formal analysis. **Mari Somerville:** Writing – review & editing, Project administration, Formal analysis, Data curation. **Lori Wozney:** Writing – review & editing, Project administration, Methodology, Funding acquisition, Data curation, Conceptualization. **Christine Cassidy:** Writing – review & editing, Methodology, Funding acquisition, Data curation, Conceptualization. **Alannah Delahunty-Pike:** Writing – review & editing, Project administration. **Rebecca Mackay:** Writing – review & editing, Project administration, Funding acquisition, Conceptualization. **Shannon MacPhee:** Writing – review & editing, Methodology, Funding acquisition, Conceptualization. **Emma Burns:** Writing – review & editing, Funding acquisition, Data curation, Conceptualization. **Helen Wong:** Writing – review & editing, Project administration, Funding acquisition, Conceptualization. **Melanie Doyle:** Writing – review & editing, Methodology, Funding acquisition, Data curation, Conceptualization. **Amy Plint:** Writing – review & editing, Resources, Methodology, Funding acquisition, Conceptualization. **Roger Zemek:** Writing – review & editing, Investigation, Funding acquisition, Data curation, Conceptualization.

## Declaration of competing interest

The authors declare the following financial interests/personal relationships which may be considered as potential competing interests: Janet Curran reports financial support was provided by Canadian Institutes of Health Research. If there are other authors, they declare that they have no known competing financial interests or personal relationships that could have appeared to influence the work reported in this paper.
